# Supplementary material for: Regulatory Efficacy of Spirulina platensis Protease Hydrolyzate on Lipid Metabolism and Gut Microbiota in High-Fat Diet-Fed Rats
Source: Int J Mol Sci. 2018 Dec 13;19(12):4023. doi: 10.3390/ijms19124023 (PMC6320850; doi:10.3390/ijms19124023)
Supplement: Supplementary file 1 [file ijms-19-04023-s001.zip › R2 revised back supplementary-sorted/ijms-388692-supplmentary figures.docx]

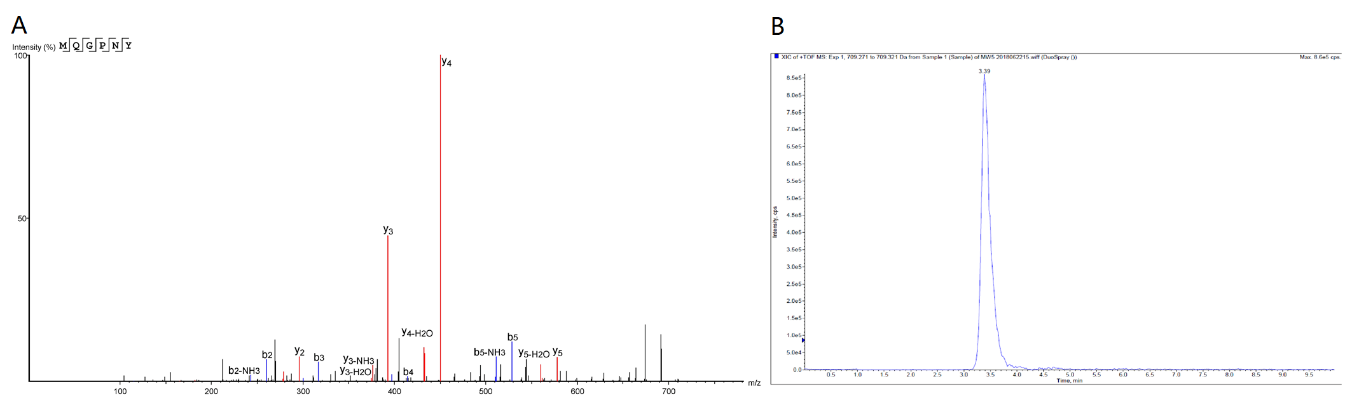


**Figure S1.** Representative chromatograms and MS/MS spectra of high abundance peptide (MQGPNY). A: MS/MS spectra of high abundance peptide, B: Representative chromatograms.


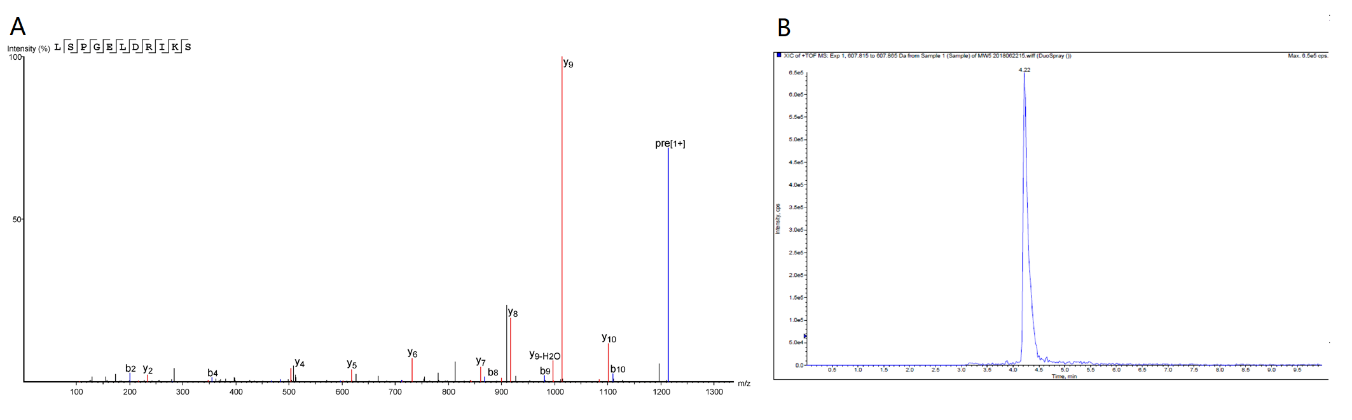


**Figure S2.** Representative chromatograms and MS/MS spectra of high abundance peptide (LSPGELDRIKS). A: MS/MS spectra of high abundance peptide; B: Representative chromatograms.


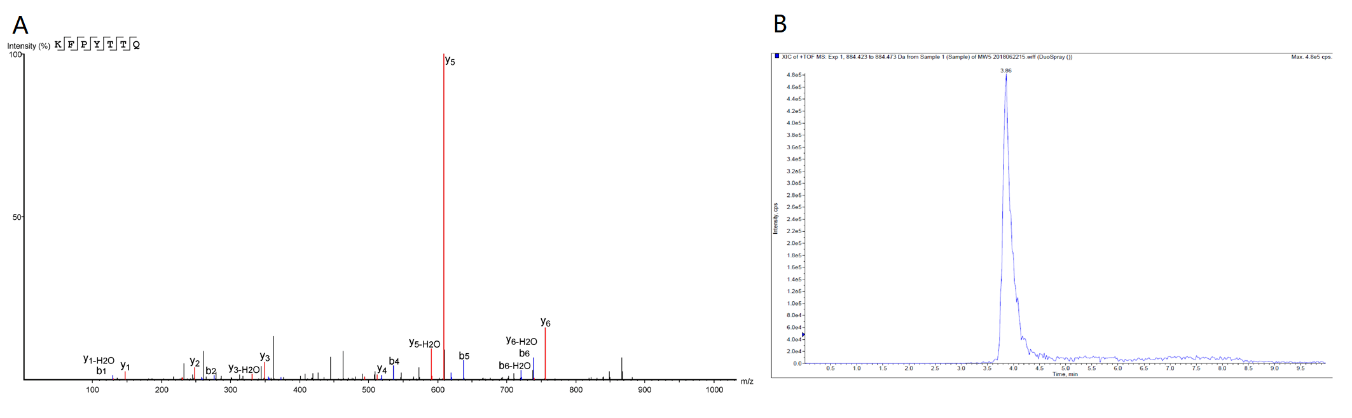


**Figure S3.** Representative chromatograms and MS/MS spectra of high abundance peptides (KFPYTTQ). A: MS/MS spectra of high abundance peptide; B: Representative chromatograms.


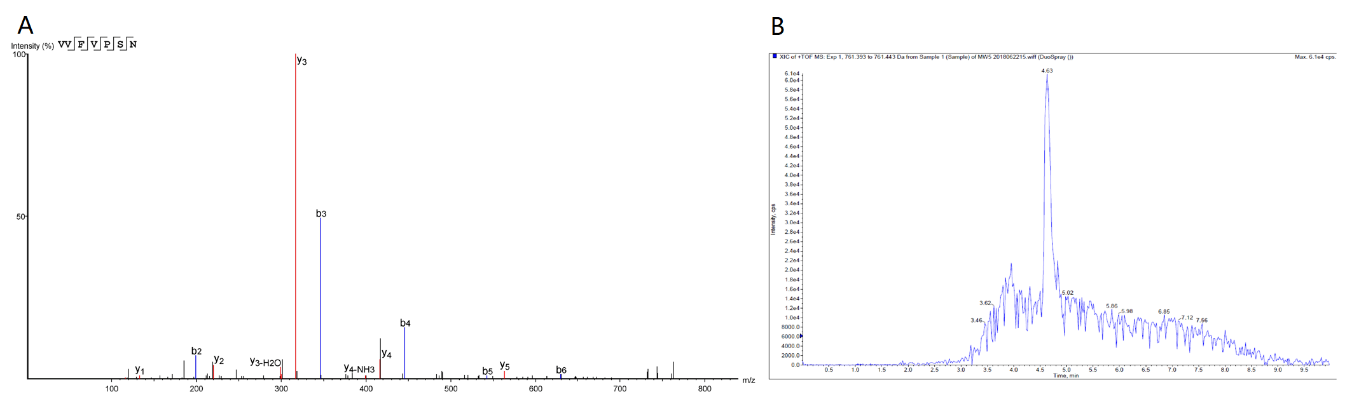


**Figure S4.** Representative chromatograms and MS/MS spectra of high abundance peptides (VVFVPSN). A: MS/MS spectra of high abundance peptide; B: Representative chromatograms.


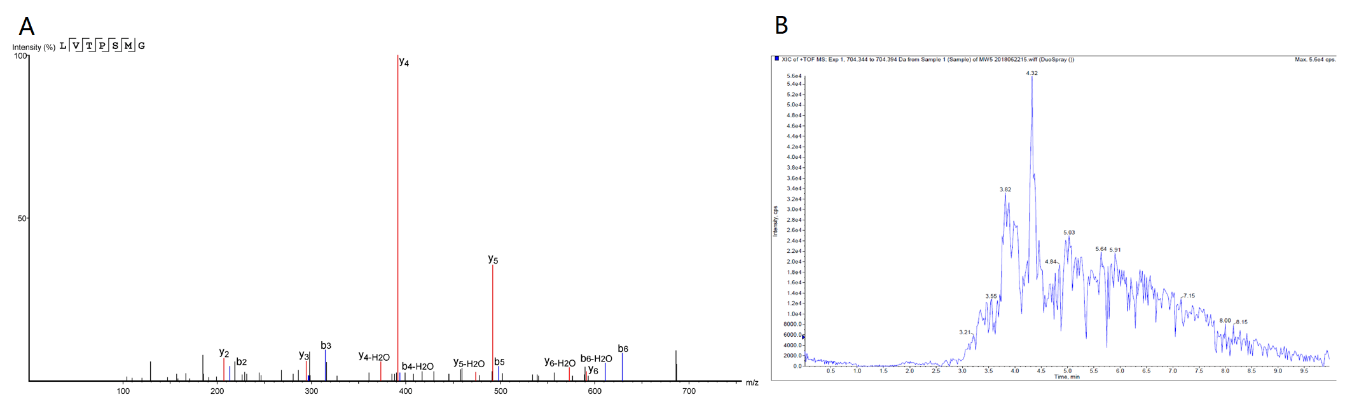


**Figure S5.** Representative chromatograms and MS/MS spectra of high abundance peptides (LVTPSMG). A: MS/MS spectra of high abundance peptide; B: Representative chromatograms.


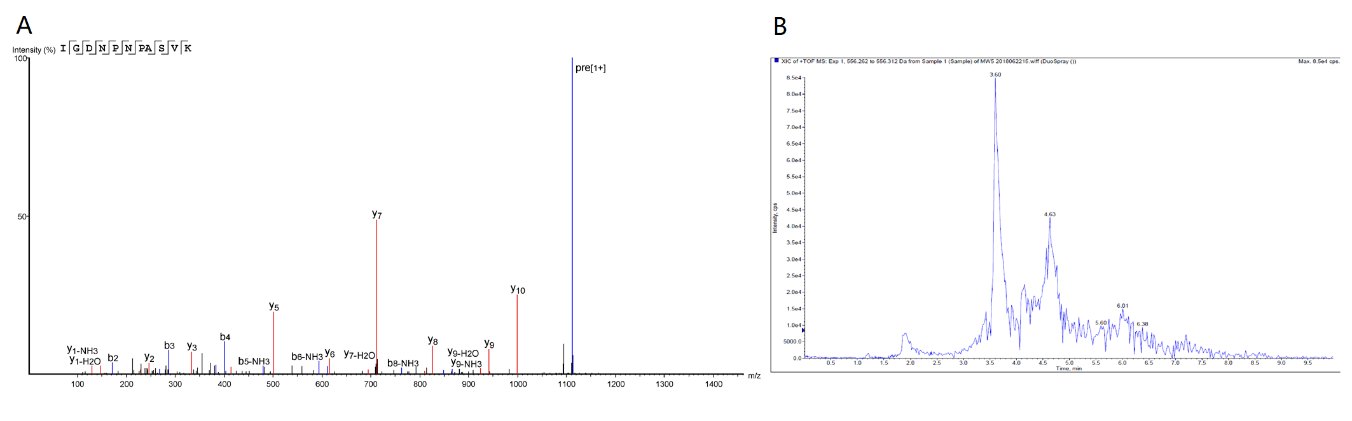


**Figure S6.** Representative chromatograms and MS/MS spectra of low abundance peptides (IGDNPNPASVK). A: MS/MS spectra of low abundance peptide; B: Representative chromatograms.


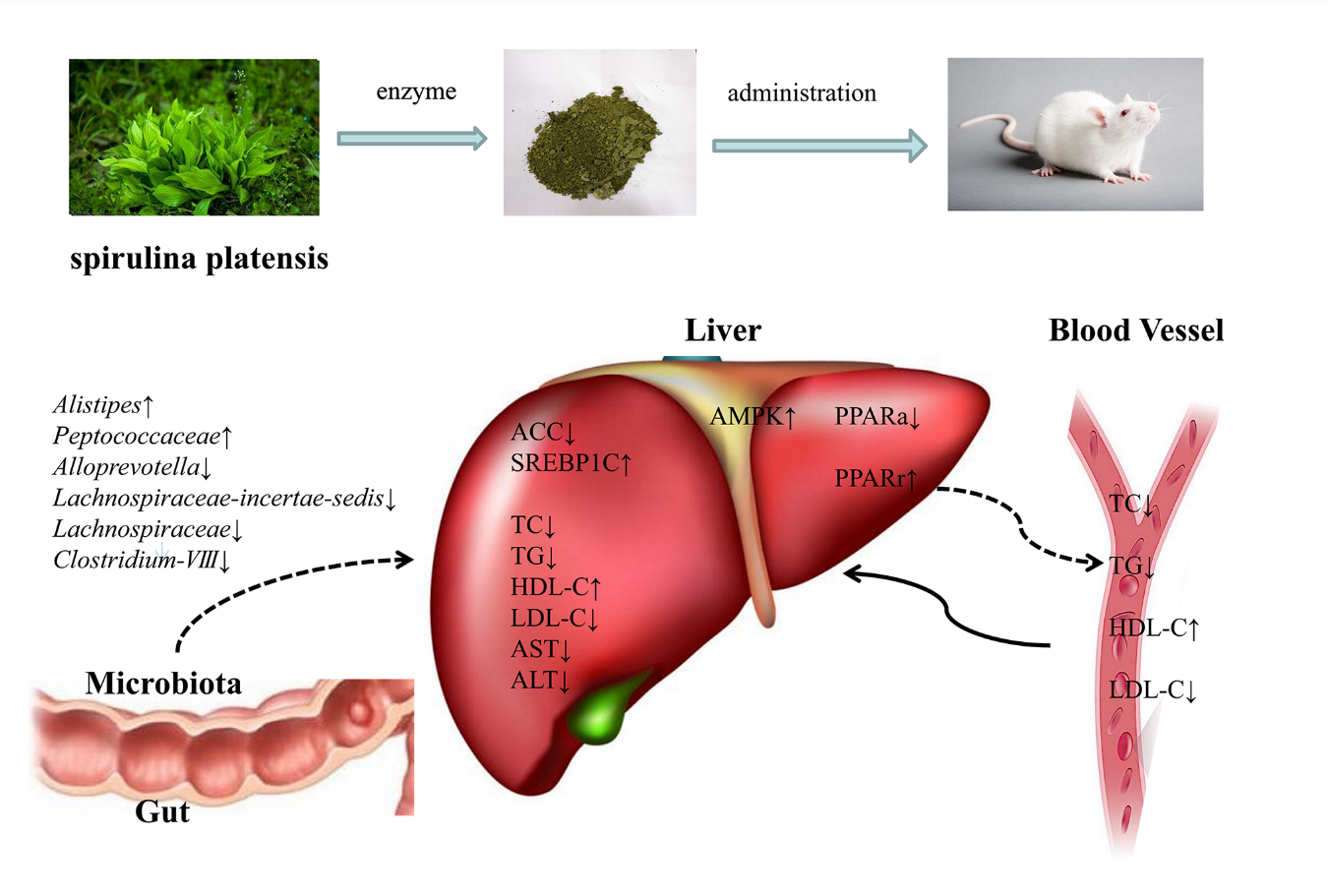


**Figure S7.** Summary of the mechanism underlying the preventive effects of SPPH on LMD.
